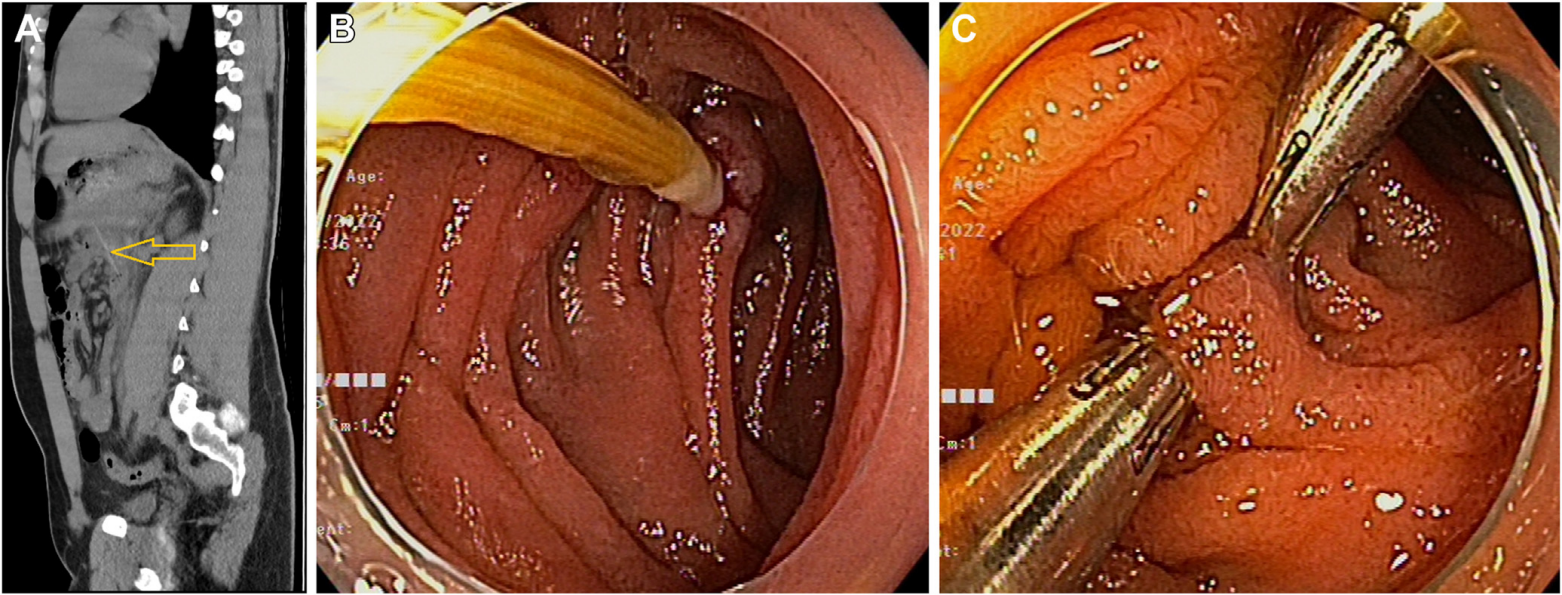# A Picky Eater: Endoscopic Removal of a Penetrating Ingested Toothpick

**DOI:** 10.1016/j.gastha.2024.07.010

**Published:** 2024-07-23

**Authors:** Nicholas Smith, Emma Davis, Daniel Burger

**Affiliations:** Department of Gastroenterology and Hepatology, Princess Alexandra Hospital, Brisbane, Queensland, Australia

A 21-year-old man presented with abdominal pain and fevers, 5 days following accidental ingestion of a toothpick. Computed tomography revealed a toothpick penetrating at the level of the duodenojejunal flexure with retroperitoneal phlegmonous changes (Figure A). Endoscopy showed a toothpick at the duodenojejunal flexure penetrating the small bowel wall (Figure B). Removal was accomplished with endoscopic cap and rat-toothed forceps, and the intestinal defect was closed using 2 clips (Figure C). The patient had rapid clinical improvement and was discharged after 5 days without operative intervention.

Toothpicks account for approximately 6.9% of ingested foreign bodies and are associated with significant mortality. The presentation of ingested toothpicks varies according to the site of impaction which most commonly occurs in the large bowel followed by the duodenum. Up to 50% of patients are unable to recall having swallowed the offending toothpick. Whilst surgical removal is traditionally used for penetrating foreign bodies, the retroperitoneal duodenum is difficult to access laparoscopically due to its deep location and limited field view resulting in a high operative morbidity. Endoscopic removal of penetrating sharp ingested foreign bodies in the duodenum can be a feasible, safe, and an effective method of removal, thereby avoiding surgical intervention in most cases.

**Figure:**